# Distribution of Posterior Corneal Astigmatism According to Axis Orientation of Anterior Corneal Astigmatism

**DOI:** 10.1371/journal.pone.0117194

**Published:** 2015-01-27

**Authors:** Toshiyuki Miyake, Kimiya Shimizu, Kazutaka Kamiya

**Affiliations:** Department of Ophthalmology, University of Kitasato School of Medicine, Kanagawa, Japan; Medical College of Soochow University, CHINA

## Abstract

**Purpose:**

To investigate the distribution of posterior corneal astigmatism in eyes with with-the-rule (WTR) and against-the-rule (ATR) anterior corneal astigmatism.

**Methods:**

We retrospectively examined six hundred eight eyes of 608 healthy subjects (275 men and 333 women; mean age ± standard deviation, 55.3 ± 20.2 years). The magnitude and axis orientation of anterior and posterior corneal astigmatism were determined with a rotating Scheimpflug system (Pentacam HR, Oculus) when we divided the subjects into WTR and ATR anterior corneal astigmatism groups.

**Results:**

The mean magnitudes of anterior and posterior corneal astigmatism were 1.14 ± 0.76 diopters (D), and 0.37 ± 0.19 D, respectively. We found a significant correlation between the magnitudes of anterior and posterior corneal astigmatism (Pearson correlation coefficient r = 0.4739, P<0.001). In the WTR anterior astigmatism group, we found ATR astigmatism of the posterior corneal surface in 402 eyes (96.6%). In the ATR anterior astigmatism group, we found ATR posterior corneal astigmatism in 82 eyes (73.9%). Especially in eyes with ATR anterior corneal astigmatism of 1 D or more and 1.5 D or more, ATR posterior corneal astigmatism was found in 28 eyes (59.6%) and 9 eyes (42.9%), respectively.

**Conclusions:**

WTR anterior astigmatism and ATR posterior astigmatism were found in approximately 68% and 91% of eyes, respectively. The magnitude and the axis orientation of posterior corneal astigmatism were not constant, especially in eyes having high ATR anterior corneal astigmatism, as is often the case in patients who have undergone toric IOL implantation.

## Introduction

In the surgical correction of astigmatism, it is important to accurately determine the magnitude and the axis orientation of the corneal astigmatism. Since the amount of anterior corneal astigmatism is far larger than that of posterior corneal astigmatism, the anterior corneal surface plays a more vital role in astigmatic correction than the posterior corneal surface. [1] Recently, the development of new technologies, such as slit-scanning devices, Scheimpflug devices, and optical coherence tomography, has made possible the quantitative measurement of the posterior corneal curvature in a clinical setting. [2–15] Moreover, a new toric intraocular lens (IOL) nomogram has been recently proposed because of the presence of posterior corneal astigmatism. [16] However, there have so far been no quantitative studies on the distribution of posterior corneal astigmatism that take the axis orientation of anterior corneal astigmatism into consideration. Since the magnitude and the axis orientation of anterior corneal astigmatism are easier to determine with the daily use of an autokeratometer or a manual keratometer, this evaluation of posterior corneal astigmatism according to the individual axis orientation of anterior corneal astigmatism may give us deep insights into astigmatic correction, especially when toric IOL implantation is performed. The purpose of this study is to retrospectively assess the distribution of posterior corneal astigmatism according to the axis orientation of anterior corneal astigmatism in a large cohort of healthy subjects.

## Patients and Methods

### Study Population

We retrospectively examined six hundred eight eyes of 608 subjects (275 men and 333 women) with good quality scans of corneal tomography measured with a Scheimpflug anterior segment photography system (Pentacam HR, Oculus, Wetzlar, Germany). The mean subject age was 55.3 ± 20.2 years (range: 15 to 96 years). Eyes with keratoconus were excluded from the study by using the keratoconus screening test of Placido disk videokeratography (TMS-5, Tomey, Nagoya, Japan). Eyes with other corneal diseases, previous ocular trauma or surgery, and contact lens use within two weeks of the measurements were also excluded. This retrospective review of the data was approved by the Institutional Review Board at Kitasato University and followed the tenets of the Declaration of Helsinki. Our Institutional Review Board waived the requirement for informed consent for this retrospective study.

### Assessment of Corneal Astigmatism

The magnitude and the axis orientation of anterior and posterior corneal astigmatism within the central 3.0-mm were automatically measured with the Scheimpflug system (Pentacam HR). This device collects 25,000 true elevation data points, which are processed to generate a 3-dimensional representation of the anterior eye. [17–19] It has been shown that this device provides corneal curvature measurements with excellent repeatability. [2, 20] We took at least three measurements and used the average value for statistical analysis. In addition, to confirm the repeatability of the measurements, the posterior corneal astigmatism measurements were made at the same time of day on 2 consecutive days in 20 eyes. The repeatability of the 2 measurements was evaluated using Bland-Altman plots, as described previously. [21] We classified astigmatism as “with-the-rule” (WTR) when the steep meridian on the corneal surface was between 60 and 120 degrees, and as “against-the-rule” (ATR) when the steep meridian on the corneal surface was between 0 and 30 degrees or between 150 and 180 degrees. Since the dioptric power of the posterior corneal surface was negative, we classified posterior corneal astigmatism as WTR when the steep meridian on the corneal surface was between 0 and 30 degrees or between 150 and 180 degrees, and as ATR when the steep meridian on the corneal surface was between 60 and 120 degrees. We classified the remaining astigmatism as oblique astigmatism. [9, 10]

## Results

**Fig 1:**
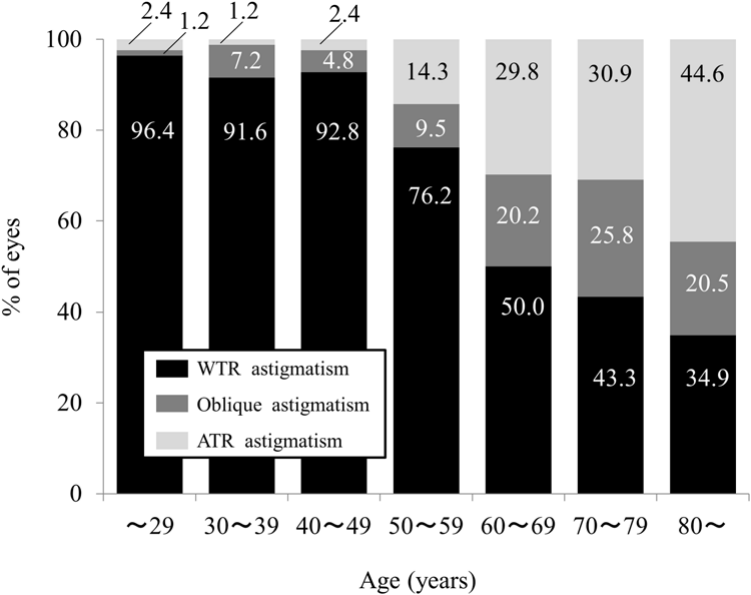
Distribution of anterior corneal astigmatism in each age group.

**Fig 2:**
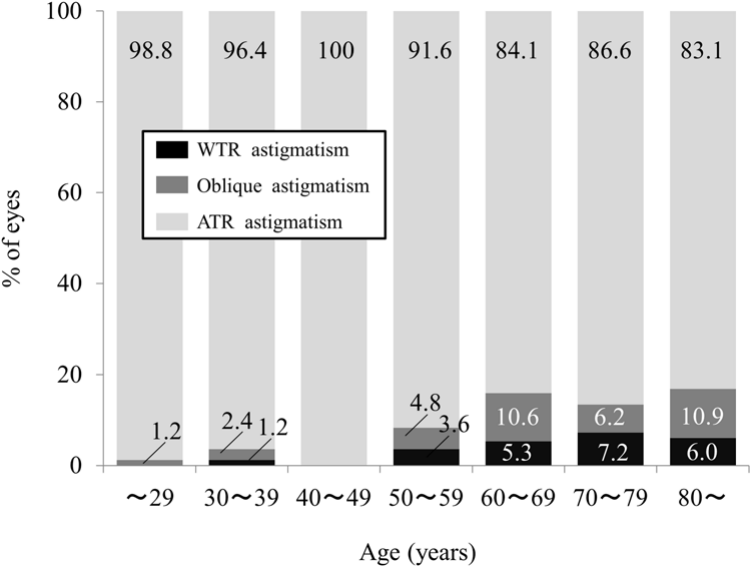
Distribution of posterior corneal astigmatism in each age group.

The demographics of the study population are summarized in Table 1. The numbers of eyes in the age groups were as follows: 18 to 29 year-old group, 84, 30 to 39 year-old group, 83; 40 to 49 year-old group, 83; 50 to 59 year-old group, 84; 60 to 69 year-old group, 94; 70 to 79 year-old group, 97; and group of 80—year-olds and over, 83 eyes. The mean magnitudes of anterior and posterior corneal astigmatism were 1.14 ± 0.76 diopters (D) (range: 0 to 4.90 D), and 0.37 ± 0.19 D (range: 0 to 1.20 D), respectively. With-the-rule (WTR) astigmatism of the anterior corneal surface was found in 68.4% (416 eyes), while against-the-rule (ATR) astigmatism of the posterior corneal surface was found in 91.3% (555 eyes) of the entire study population. We found a significant correlation between the magnitudes of anterior and posterior corneal astigmatism (Pearson correlation coefficient r = 0.4739, P<0.001). Figs. 1 and 2 show the respective distributions of anterior and posterior corneal astigmatism in each age group. We found a tendency for a high prevalence of ATR anterior corneal astigmatism with aging, whereas most eyes in all age groups showed ATR posterior corneal astigmatism.

**Table 1:** Demographics of the study population.

**Demographic data and results**	
Subjects	608
Age (years)	55.3 ± 20.2 years (range, 15 to 96 years)
Gender (Male: Female)	M: F = 275: 333
Anterior corneal astigmatism (D)	1.14 ± 0.76 D (range, 0 to 4.9 D)
Posterior corneal astigmatism (D)	0.37 ± 0.19 D (range, 0 to 1.2 D)

D = diopter

**Fig 3:**
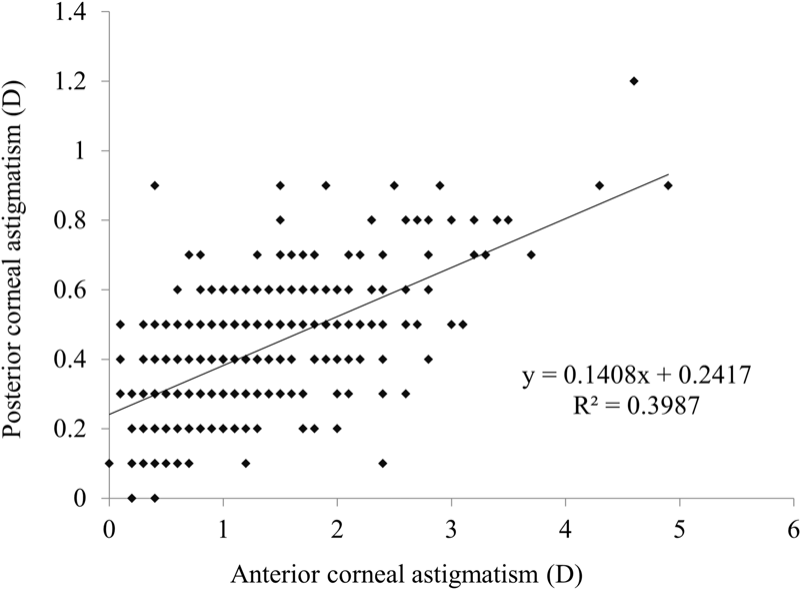
A graph showing a significant correlation between the magnitudes of anterior and posterior corneal astigmatism (Pearson correlation coefficient r = 0.5746, P<0.001) in eyes with-the-rule (WTR) anterior corneal astigmatism.

**Fig 4:**
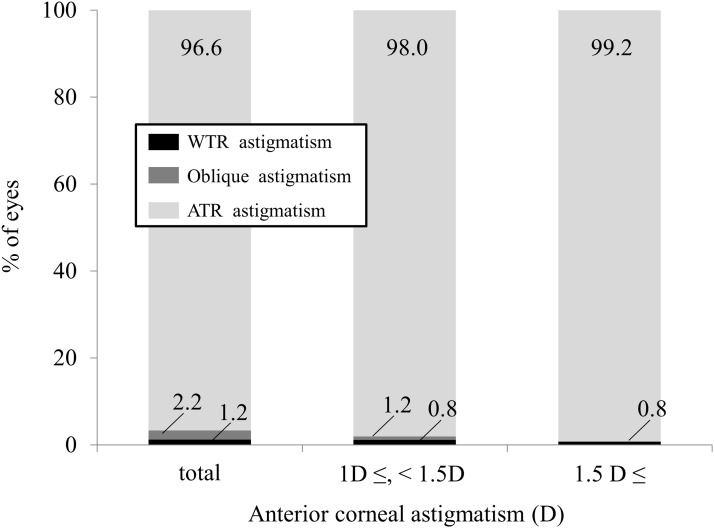
The axis orientation of posterior corneal astigmatism in eyes with with-the-rule (WTR) anterior corneal astigmatism.

In the WTR astigmatism of the anterior corneal surface group, 402 eyes (96.6%) showed ATR astigmatism of the posterior corneal surface. There was a significant correlation between the magnitudes of anterior and posterior corneal astigmatism (r = 0.5746, P<0.001) (Fig. 3). In eyes with WTR astigmatism of 1 D and 1.5 D or more, 248 eyes (98.0%) and 131 eyes (99.2%), respectively, showed ATR astigmatism of the posterior corneal surface (Fig. 4).

**Fig 5:**
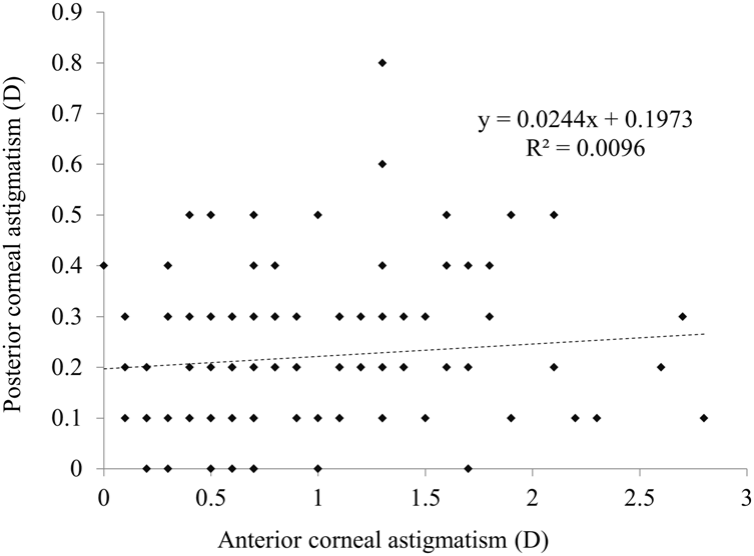
A graph showing no significant correlation between the magnitudes of anterior and posterior corneal astigmatism (Pearson correlation coefficient r = 0.1010, P = 0.2918) in eyes against-the-rule (ATR) anterior corneal astigmatism.

**Fig 6:**
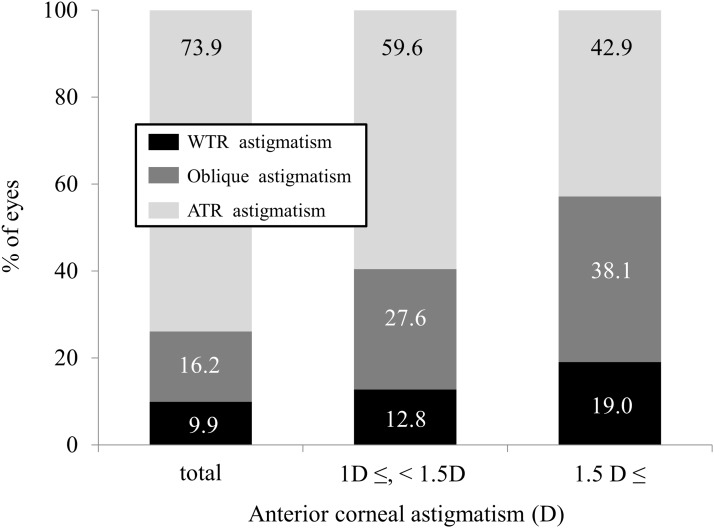
The axis orientation of posterior corneal astigmatism in eyes with against-the-rule (ATR) anterior corneal astigmatism.

In the ATR astigmatism of the anterior corneal surface group, 82 eyes (73.9%) showed ATR astigmatism of the posterior corneal surface. There was no significant correlation between the magnitudes of anterior and posterior corneal astigmatism (r = 0.1010, P = 0.2918) (Fig. 5). In eyes with ATR astigmatism of 1 D and 1.5 D or more, 28 eyes (59.6%) and 9 eyes (42.9%), respectively, showed ATR astigmatism of the posterior corneal surface (Fig. 6).

**Fig 7:**
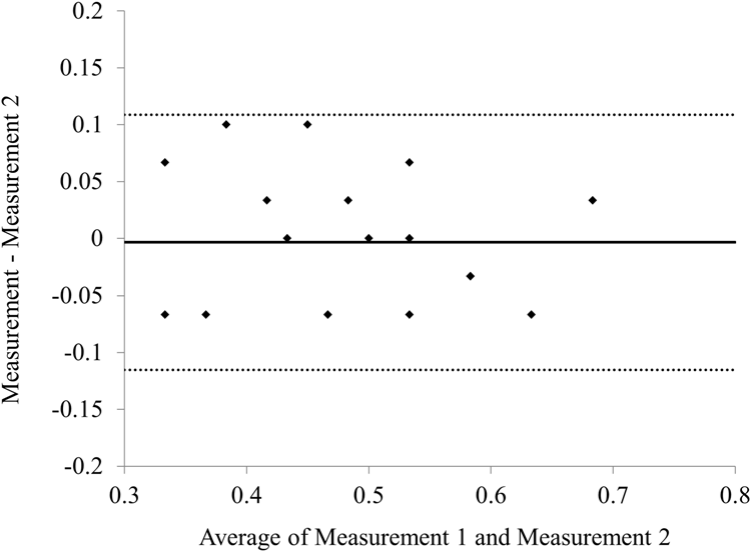
Bland-Altman plots shows the difference between 2 measurements divided by mean of these posterior corneal astigmatism measurements. The solid lines represent mean differences between 2 consecutive measurements of posterior corneal astigmatism, dotted lines are the upper and lower borders of the 95% LoA (mean difference ± 1.96 multiplied by SD of the mean difference).

Bland-Altman plots indicate that the mean difference between 2 measurements with the Scheimpflug system (±95% limits of agreement [LoA]) was-0.003 ± 0.057 D (range-0.007 to 0.100 D) (Fig. 7).

## Discussion

In the present study, our results demonstrated that approximately 68% of the eyes of the entire study population had WTR astigmatism of the anterior corneal surface, and that approximately 91% of eyes had ATR astigmatism of the posterior corneal surface. These ATR findings were in line with previous findings of posterior corneal astigmatism, as shown in Table 2. [3–9, 13] Moreover, our results also showed a tendency for a high prevalence of ATR anterior corneal astigmatism with aging, whereas most eyes showed ATR posterior corneal astigmatism in all age groups, which was in agreement with previous reports on age-related changes in corneal astigmatism. [8–10] The mean change in ATR astigmatism of the anterior corneal surface (0.18 D in 5 years) was far greater than that in WTR astigmatism of the posterior corneal surface (0.02 D in 5 years), [10] suggesting that the shape of the posterior corneal surface was relatively unchanged by aging.

**Table 2:** Published values of posterior corneal astigmatism.

**Study**	**Imaging Modality Used**	**Eyes/ Patients**	**Age (years)**	**Mean magnitude of posterior corneal astigmatism (D)**	**Range of posterior corneal astigmatism (D)**
Royston JM ^3^	Purkinje image (Polaroid camera)	5/5	-	0.38	0.17–0.78
Dunne MC ^4^		60/60	22.0±3.3	0.26	-
Prisant O ^5^	Scanning-slit topography (Bausch & Lomb Orbscan)	40/31	-	0.66±0.23	0.32–1.38
Módis L Jr ^6^		44/44	61.4±16.4	0.78±0.61	0.16–3.3
Dubbelman M ^7^	Scheimpflug photography in 6 fixed meridians (Topcon SL-45 camera)	114/114	39±14	0.305	-
Koch DD ^9^	Rotating Scheimpflug imaging (Ziemer Galilei DSA)	715/435	55±20	0.30±0.15	0.01–1.1
Wang L ^13^		20/20	36±12.5	0.25±0.08	0.10–0.42
Ho JD ^8^	Rotating Scheimpflug imaging (Oculus Pentacam)	493/493	41.1±21.9	0.33±0.16	0–0.94
Current		608/608	55.3±20.2	0.37±0.19	0–1.2

D:diopter

There have been only a few previous studies on the relationship of the axis orientation of posterior corneal astigmatism with aging, [9, 10] and no studies on its relationship with aging which especially considered the magnitude of the astigmatism. As far as we can ascertain, this is the first study to assess the relationships of both the magnitude and the axis orientation of corneal astigmatism with aging in a large cohort of healthy subjects.

In the WTR astigmatism group, 96.6% of eyes showed ATR posterior corneal astigmatism, and there was a significant correlation between the magnitudes of anterior and posterior corneal astigmatism. Even when the magnitude of anterior corneal astigmatism was increased, ATR posterior corneal astigmatism was seen in 96% of eyes or more, suggesting that ATR posterior corneal astigmatism remained unchanged in eyes with WTR anterior corneal astigmatism. On the other hand, in the ATR astigmatism group, 73.9% of eyes showed ATR posterior corneal astigmatism, and there was no significant correlation between the magnitudes of anterior and posterior corneal astigmatism. Especially when the magnitude of anterior corneal astigmatism was increased, the percentage of ATR posterior corneal astigmatism was considerably decreased.

In eyes with WTR astigmatism of the anterior corneal surface, the presence of ATR astigmatism of the posterior corneal surface compensates for the anterior corneal astigmatism, and thus reduces the total corneal astigmatism. However, in eyes with ATR astigmatism of the anterior corneal surface, it increases total corneal astigmatism. Accordingly, a slight undercorrection in eyes with WTR anterior corneal astigmatism and a slight overcorrection in eyes with ATR anterior corneal astigmatism is recommended for successful toric IOL implantation, based on previous findings that most eyes show ATR posterior corneal astigmatism. [9, 10, 16] Based on our current findings of anterior and posterior corneal astigmatism, when toric IOL implantation is planned for eyes with WTR anterior corneal astigmatism, a slight undercorrection of astigmatism is desirable in order to accurately correct total corneal astigmatism, because most eyes showed the ATR posterior corneal astigmatism. However, a slight overcorrection is not always appropriate, and individual assessment of posterior corneal astigmatism is necessary, especially when toric IOL implantation is planned for eyes with high ATR anterior corneal astigmatism. We believe that, although our findings are simple, they are clinically meaningful for successful toric IOL implantation for the correction of astigmatism. It should be noted that the magnitude and the axis of posterior corneal astigmatism were not constant, especially when we corrected high ATR astigmatism of the anterior corneal surface—as is often the case with toric IOL implantation.

There are at least two limitations to this study. One is that it was conducted in a retrospective fashion. A randomized, controlled study may provide further information for confirming the authenticity of these results. Another limitation is that we determined the magnitude and the axis orientation of posterior corneal astigmatism only using the Scheimpflug system. Our results need to be validated by more rigorous analysis using another methods. However, it has been demonstrated that the device has an excellent repeatability of the corneal curvature measurements. [2, 20] Moreover, as shown in Fig. 7, we confirmed the good repeatability of the posterior corneal astigmatism measurements in the current study, as evidenced by the narrow 95% LoA. Hence, we believe that this device offers reasonable repeatability in the clinical evaluation of posterior corneal astigmatism.

In conclusion, our results support the view that approximately 68% and 91% of eyes show WTR anterior corneal astigmatism and ATR posterior corneal astigmatism, respectively, and that the prevalence of ATR posterior corneal astigmatism is decreased as the magnitude of ATR anterior corneal astigmatism is increased. The magnitude and the axis orientation of posterior corneal astigmatism were not constant, especially in eyes having high ATR astigmatism of the anterior corneal surface, as is often the case with toric IOL implantation. We believe that, despite the simplicity of our findings, they are helpful for achieving successful toric IOL implantation for the correction of astigmatism in a clinical setting.
